# Improvement in Transient Agarose Spot (TAS) Cell Migration Assay: Microplate-Based Detection and Evaluation

**DOI:** 10.3390/ijms26125584

**Published:** 2025-06-11

**Authors:** Apor Veres-Székely, Csenge Szász, Domonkos Pap, Péter Bokrossy, Dorina Lenzinger, Tamás Visnovitz, Judith Mihály, Marcell Pálmai, Zoltán Varga, László Őrfi, Attila J. Szabó, Ádám Vannay, Beáta Szebeni

**Affiliations:** 1 Pediatric Center, Semmelweis University, 1083 Budapest, Hungary; 2 HUN-REN-SU Pediatrics and Nephrology Research Group, 1052 Budapest, Hungary; 3 Institute of Genetics, Cell and Immunobiology, Semmelweis University, 1089 Budapest, Hungary; 4 Department of Plant Physiology and Molecular Plant Biology, ELTE Eötvös Loránd University, 1117 Budapest, Hungary; 5 TTK Biological Nanochemistry Research Group, Institute of Materials and Environmental Chemistry, Research Centre for Natural Sciences, 1117 Budapest, Hungary; 6 Department of Physical Chemistry and Materials Science, Faculty of Chemical Technology and Biotechnology, Budapest University of Technology and Economics, 1111 Budapest, Hungary; 7 Vichem Chemie Research Ltd., 1022 Budapest, Hungary

**Keywords:** cell migration, TAS assay, microplate reader, Hoechst

## Abstract

Collective cell migration is crucial in various biological processes, including tumor progression and metastasis. The widely used scratch assay (wound healing assay) has limitations in throughput, reproducibility, and data analysis. To overcome these challenges, we previously developed the Transient Agarose Spot (TAS) assay, which enhanced assay precision and reproducibility. In this study, we present an improved microplate-based TAS assay. By using a microplate reader, we automated data acquisition, enabling the detection of cell migration in a 96-well plate format with greater throughput and accuracy. The new method applies Hoechst staining to label viable cells, providing a stable signal for kinetic analysis without compromising cell viability. We validated this approach with fluorophore-expressing cancer cells and demonstrated its ability to monitor dose-dependent effects of fetal bovine serum on cell migration. Additionally, we applied the microplate-based TAS assay to assess the anti-migratory effects of kinase inhibitors and mesenchymal stem cell-derived extracellular vesicles (EVs) on lung cancer cells. The assay accurately quantified migration inhibition and revealed the concentration-dependent effects of EVs, highlighting their potential as therapeutic agents. This microplate-based TAS assay provides a scalable, efficient, and cost-effective platform for high-throughput screening of cell migration and drug discovery, offering a robust alternative to traditional microscopy-based methods.

## 1. Introduction

Collective cell migration is a fundamental biological process that plays a crucial role in various physiological and pathological conditions, including tumor progression [1]. The ability of cancer cells to migrate and invade surrounding tissues is a hallmark of malignancy and a major determinant of prognosis [2]. Understanding the mechanisms of cancer cell migration is essential for identifying therapeutic targets and developing novel treatment strategies aimed at limiting metastasis. While traditional microscopy-based methods, particularly the widely used scratch assay, have been extensively used to study cell migration, their inherent limitations, such as low throughput and labor-intensive protocols, restrict their utility in both basic and applied cancer research [3,4,5]. In our previous work, we developed the Transient Agarose Spot (TAS) cell migration assay, which offered enhanced analytical precision and reproducibility compared to the gold-standard scratch assay [4]. Although most limitations of the scratch assay were eliminated, data acquisition still relied on microscopy, requiring expensive equipment systems and time-consuming manual work for high-throughput screening.

As phenotypic readouts such as cell migration offer integrative biological insight, phenotype-based cellular assays have gained prominence in early-stage drug discovery. These assays are increasingly recognized as valuable tools in preclinical drug development, particularly for their ability to capture complex biological responses beyond single-target effects. These assays support untargeted screening, enabling the discovery of novel mechanisms of action and phenotypic effects that might be overlooked by target-based approaches [6,7]. They are widely used for hit identification, triaging, and lead optimization, and their effectiveness depends on thoughtful assay design, the use of appropriate chemical libraries, and robust strategies for hit validation [8].

Here, we present an improved version of the TAS assay, transitioning from microscopy-based detection to a microplate-based system. This new approach significantly enhances the throughput and efficiency of the cell migration assay while preserving its sensitivity, reliability, and accuracy.

## 2. Results

### 2.1. Data Acquisition by Microplate Reader

**Figure 1:**
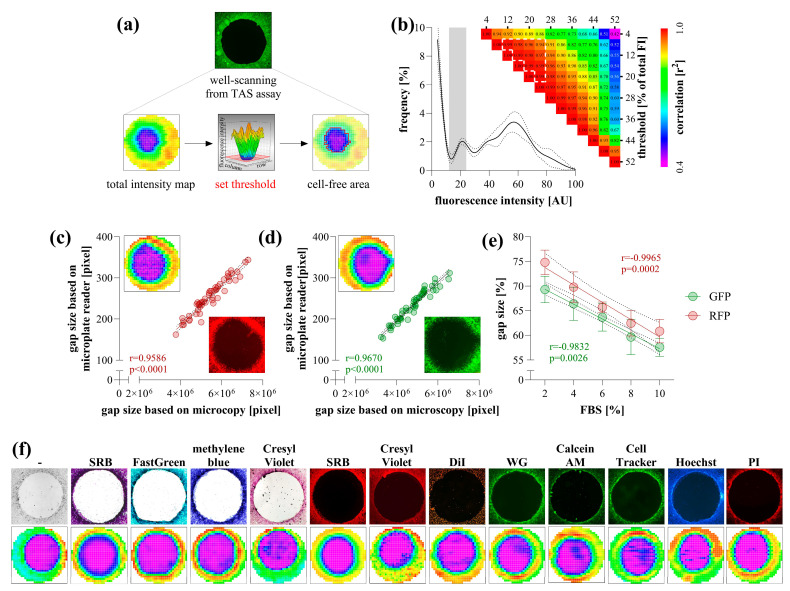
Data acquisition of transient agarose spot (TAS) assay using microplate reader. The well-scanning mode of the microplate reader generates an intensity heatmap of a whole well, where the cell-free area can be defined by setting a threshold value (**a**). The accuracy of threshold selection was evaluated using TAS assay wells containing GFP-expressing HCT-116 cells (n = 50) (**b**). Based on the intensity distribution of heatmap pixels, different threshold values were selected to determine cell-free areas, and a correlation matrix was generated. A nearly perfect correlation was observed for threshold values between 10 and 25% of the maximal intensity (highlighted with white outline). Validation of the microplate-based detection was carried out by correlating gap sizes with microscopy-based measurements for RFP- (**c**) and GFP-expressing (**d**) HCT-116 cells (n = 50). The applicability of microplate reader-based quantification was investigated on RFP- and GFP-expressing HCT-116 cells after their stimulation with a dilution series of fetal bovine serum (FBS) for 72 h (n = 10/group) (**e**). Various colorimetric and fluorescent stains were tested on non-fluorescent HCT-116 cells (**f**). The same wells were captured by microscopy (top, using 4× magnification) and microplate reader (bottom). Heatmaps represent well-scanning fluorescence intensity data, where color gradients correlate with signal strength across the scanned area. Significance values (*p*) and Pearson’s correlation coefficients (r) are indicated.

To determine the area of cell-free zones, the wells of the TAS assay containing stained cells were detected using the "well scanning" mode of the microplate reader. The resulting heatmap comprised 708 scanning points, capturing a data range of absorbance or fluorescence that transitions sharply from the cell-free area to full confluency. The area of cell-free zones was determined by setting a threshold value to define the edge of the examined area and by quantifying the points below this threshold (Figure 1a). The optimal threshold was between 10 and 25% of the maximal intensity values, within which the resulting cell-free areas exhibited a perfect correlation with each other (Figure 1b).

To validate the detection method, an entire TAS assay plate containing RFP- and GFP-expressing HCT-116 cells was scanned using both a microplate reader and microscopy (Figure 1c,d). The calculated areas showed perfect correlation between the two methods. Furthermore, microplate-based detection of RFP- and GFP-expressing HCT-116 cells stimulated with a dilution series of FBS yielded linear dose–response relationships with very high correlation coefficients (Figure 1e).

Non-fluorescent cells were first scanned in their native, unstained state using a microplate reader by measuring absorbance in the UV range. However, detection was only reliable in a medium-free environment, requiring the replacement of the culture medium with PBS. To enhance visualization, various colorimetric and fluorescent staining methods were applied, all of which were detectable with a microplate reader (Figure 1f). These methods provided clear visualization, allowing the edge of the cell-free area to be precisely defined.

### 2.2. Optimization of Hoechst Staining

**Figure 2:**
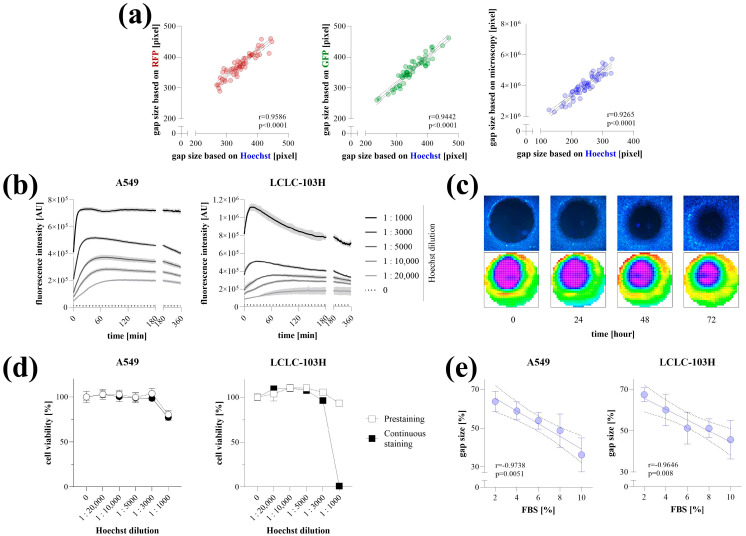
Optimization of Hoechst-staining. Validation of Hoechst staining for microplate-based detection was carried out in fluorescent and native HCT-116 cells (**a**). Gap areas determined by Hoechst fluorescence intensity (pre-stained cells for 4 h at 1:10,000 concentration) were correlated with RFP and GFP signals, as well as microscopy-based quantification (n = 50, using 4× magnification). Staining kinetics with various Hoechst concentrations were investigated on A549 and LCLC-103H cells (n = 5/group) with two-minute interval detection (**b**). The stability of Hoechst fluorescence signal and its detection with microscope (top) or microplate reader (bottom) was investigated for 72 h (**c**). The cytotoxicity of Hoechst staining was investigated on A549 and LCLC-103H cells using MTT cell viability assay following a 4 h prestaining or continuous staining for 72 h at 1:10,000 concentration (**d**). The applicability of microplate reader-based quantification was investigated on Hoechst-stained A549 and LCLC-103H cells after their stimulation with a dilution series of fetal bovine serum (FBS) for 72 h (n = 10/group) (**e**). Heatmaps represent well-scanning fluorescence intensity data, where color gradients correlate with signal strength across the scanned area. Results are presented as mean ± SD. Significance values (*p*) and Pearson’s correlation coefficients (r) are indicated.

The applicability of Hoechst staining for microplate-based detection was verified on RFP-, GFP-expressing, and native HCT-116 colon cancer cells. The gap areas determined based on the Hoechst fluorescence signal detected by the microplate reader showed a high correlation with RFP or GFP signals, as well as the gap size quantified by microscopy (Figure 2a).

The Hoechst staining of living cells was optimized on A549 and LCLC-103H lung cancer cells. We found that even very low dye concentrations (1:20,000, 1:10,000) resulted in a stable fluorescence signal, whereas higher concentrations initially produced higher intensity, which decreased over time before finally settling at a similar value (Figure 2b). Fluorescence was detectable even 72 h after staining the cells with Hoechst at a 1:10,000 dilution for 4 h (Figure 2c). Cytotoxic effects of Hoechst staining were observed only at high concentrations, specifically when the dye-containing culture medium was not replaced after the 4 h incubation period (Figure 2d). The microplate-based detection of Hoechst-stained cells stimulated with a dilution series of FBS for 72 h yielded linear dose–response relationships with very high correlation coefficients (Figure 2e).

### 2.3. Representative Experiments

**Figure 3:**
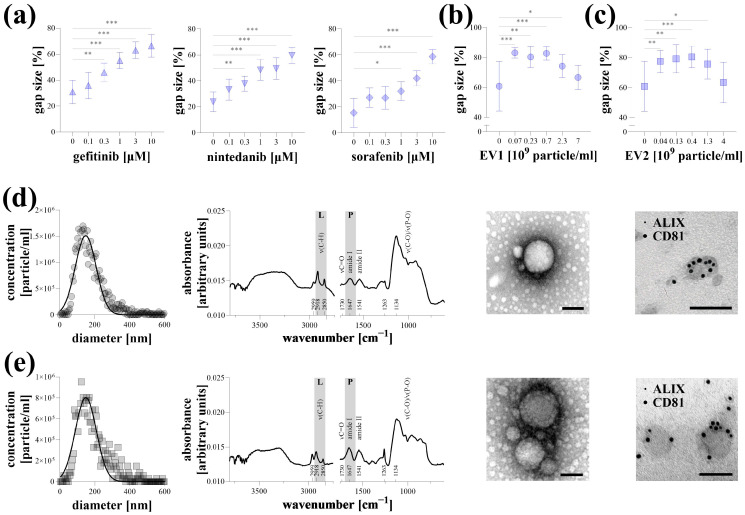
Representative experiments of microplate-based transient agarose spot (TAS) cell migration assay. Hoechst-stained A549 cells were stimulated with 10% FBS and the antimigratory effect of gefitinib, nintedanib, and sorafenib kinase inhibitors, (**a**) as well as EV1 (**b**) and EV2 (**c**) MSC-derived extracellular vesicles, was investigated after 72 h via microplate-based quantification (n = 10/group). Results are presented as mean ± SD. * *p* < 0.05; ** *p* < 0.01; *** *p* < 0.001; (Brown–Forsythe and Welch ANOVA with Dunnett’s T3 test). Characterization of EV1 (**d**) and EV2 (**e**) samples was carried out by determining nanoparticle size distribution using nanoparticle tracking analysis, measuring protein and lipid content using surface-enhanced Fourier transform infrared spectroscopy, investigating morphology and structure using transmission electron microscopy (TEM), and demonstrating the presence of ALIX1 and CD81 markers by immuno-TEM using 5 and 10 nm gold labeled antibodies. Scale bar: 100 nm.

Representative experiments were conducted on Hoechst-stained A549 lung cancer cells stimulated with 10% FBS, with data acquisition performed using a microplate reader. Kinase inhibitors, including gefitinib, nintedanib, and sorafenib, reduced cell migration in a dose-dependent manner (Figure 3a). Similarly, EV1 and EV2 extracellular vesicles, isolated from the supernatant of MSCs, inhibited the migration of A549 cells. However, this effect was diminished at high nanoparticle concentrations (Figure 3b,c). The properties of EV1 (Figure 3d) and EV2 (Figure 3e) were characterized using various analyses. Based on NTA measurements, the average particle sizes for both samples were approximately 150 nm, with a concentration of 1–2 × 10^10^ particles/mL. After buffer subtraction, surface-enhanced IR spectra of the samples revealed characteristic absorption bands of EVs, indicating a typical protein-to-lipid ratio. TEM imaging demonstrated a typical morphology of a small EV fraction. The presence of ALIX and CD81, labeled with 5 nm and 10 nm gold particles, respectively, was detectable on the surface of the EVs.

## 3. Discussion

Collective cell migration plays a crucial role in various diseases and biological processes, including embryonic development, wound healing, tumor progression, and metastasis or tissue fibrosis [9]. The scratch assay, widely considered the gold standard for studying cell migration, has several limitations, including intra- and inter-assay variability and labor-intensive data analysis [10,11,12]. To overcome these challenges, our research group previously developed the TAS cell migration assay, which eliminates the adverse effects of scratching the cell monolayer by using removable agarose droplets as physical barriers to create cell-free areas [4]. This innovation significantly improved assay efficiency and enabled its adaptation to a 96-well plate format, reducing material (both reagents and plastic) consumption and increasing assay accuracy and throughput. Although the TAS assay was developed on fibroblasts, its usability on cancer cells was also demonstrated. However, the detection method remained microscopy-based, which continued to face challenges due to its time and equipment requirements, thereby limiting overall efficiency [12].

In this study, we aimed to transfer data acquisition and analysis from microscopy to a microplate reader. New-generation microplate readers (e.g., devices of BMG LABTECH (Ortenberg, Germany), Tecan (Männedorf, Switzerland), and Molecular Devices (San Jose, CA, USA)) feature a well-scanning mode specifically designed to address non-homogeneous data from cell-based assays, where an uneven cell distribution across the well renders a single central measurement insufficient. This advanced scanning approach ensures more accurate and representative data acquisition by capturing multiple measurements within individual wells.

We utilized the CLARIOstar Plus microplate reader (BMG LABTECH, Ortenberg, Germany) combined with MARS software to scan a 4 mm diameter region at the center of each well in a 96-well plate. The area was scanned in a 30 × 30 matrix mode, generating absorbance or fluorescence intensity heatmaps with several hundred data points within the region of interest. The scanning points derived from TAS assay heatmaps clustered into two distinct groups: high absorbance or fluorescence values representing cell-rich regions, and near-blank values indicating cell-free zones. The border between these two groups was well-defined, enabling the establishment of a precise threshold value to demarcate and accurately measure the area of cell-free zones (Figure 1a,b). Although most limitations of the scratch assay were eliminated, data acquisition still relied on microscopy, requiring expensive equipment systems and time-consuming manual work, while the risk of bias from manual evaluation remained minimal.

The accuracy of data evaluation by the microplate reader was verified using fluorophore-expressing cancer cells (Figure 1c–e), where area quantification yielded identical gap sizes compared to the graphical analysis of the microscopic images from the same samples. The microplate reader-based evaluation has proven to be capable of tracking the dose-dependent effect of FBS on the migration of fluorescent cancer cells. FBS is an optimal inducer of cell migration due to the fact that it contains growth factors and chemoattractants [13,14]. In addition, as a general nutrient, it enhances overall metabolic activity, thereby promoting autocrine signalling, which leads to sustained receptor tyrosine kinase activation and cell proliferation, both of which are driving forces for cell migration [15,16]. However, genetically modified fluorophore-expressing cells are not always applicable. Although the UV absorbance of unlabeled cells could also be detected, this approach is impractical due to the high background signal originating from culture medium components such as phenol red, amino acids, and FBS [17]. A clear border of the cell-free area can only be detected in PBS. Therefore, various colorimetric and fluorescence staining methods were investigated to assess their suitability for microplate reader-based detection (Figure 1f). The applied dyes, targeting intracellular proteins, cell membrane, and DNA, as well as those activated by intracellular esterase activity, yielded a satisfactory signal-to-noise ratio.

Of all the options we tested, Hoechst staining proved to be the most suitable approach for performing the TAS cell migration assay. Hoechst is a cost-effective, cell-permeable dye that allows the labeling of viable cells without fixation or permeabilization, unlike other approaches, such as colorimetric staining or using the DNA stain propidium iodide [18]. Compared to cell fixation, which converts the assay into an end-point measurement, using a non-toxic dye that labels live cells allows for multiple detections in kinetic assays. Hoechst intensity is amplified upon DNA binding; therefore, the potential leakage of free dye molecules over time does not increase the background signal, a phenomenon commonly observed in esterase-dependent approaches [19]. The use of membrane dyes can be a good alternative; however, as they are lipophilic molecules, they can be challenging to handle in water-based cellular media, and their cost is relatively higher [20]. In addition to precise area detection after Hoechst staining, a stable signal was obtained even at low concentrations, leading to high contrast at the edges of the cell-free zones for several days, while no signs of cytotoxicity were detected (Figure 2). In summary, the migration of Hoechst-stained cancer cells following FBS induction can be reliably monitored using microplate-based detection. However, it should be noted that Hoechst may interfere with drug transporters (e.g., several ATP-binding cassette (ABC) transporters, including ABCG2/BCRP, ABCB1/P-glycoprotein/MDR1, and ABCC1/MRP1), which is an important consideration when using the TAS assay for drug screening [21,22,23]. Since these proteins are frequently implicated in multidrug resistance, particularly in cancer cells, it is advisable to evaluate whether the compound of interest interacts with the same transport pathways as Hoechst. This can be explored using public databases such as DrugBank (for known substrates or inhibitors), via in vitro transporter-binding assays, or by comparing the compound’s effective concentration in parallel experiments conducted in the absence or presence of Hoechst staining. If the tested compounds are likely to compete with Hoechst for transporter binding, using a membrane dye may be a more suitable alternative. This is supported by our previous study describing the original TAS method, where the membrane dye DiI was successfully used to label viable cells under similar experimental conditions [4]. While these offered staining procedures are generally applicable across various cell types, the intensity and kinetics of dye uptake may vary depending on the specific characteristics of each cell line. Therefore, we recommend that users perform initial optimization steps—similar to those presented here—tailored to their own experimental systems to ensure accurate and reproducible results.

Finally, in representative experiments, we demonstrated the applicability of the microplate-based TAS assay on Hoechst-stained lung cancer cells (Figure 3). Gefitinib, nintedanib, and sorafenib are kinase inhibitors widely used in the treatment of non-small-cell lung cancer [24,25,26]. As these drugs target receptor tyrosine kinases and other signal transduction pathways involved in cancer cell migration and proliferation, they serve as appropriate candidates for demonstration. In our experiment, these compounds exhibited a dose-dependent inhibitory effect on FBS-induced migration of A549 cancer cells (Figure 3a).

In recent years, the potential therapeutic role of MSC-derived EVs has emerged in various diseases, particularly in different cancers [27]. Although the reported data are not entirely consistent, several studies have demonstrated the anticancer effect of stem cell-derived EVs, particularly their ability to reduce the collective migration of cancer cells in vitro [28,29]. Similarly, we observed a significant anti-migratory effect of EVs isolated from the supernatant of MSCs (Figure 3b–e), which, surprisingly, was diminished at higher particle concentrations. The loss of the biological effect of EVs at elevated numbers has also been described previously [30]. One possible explanation for this is that an increased particle concentration promotes the aggregation of extracellular vesicles [31].

In this study, we successfully developed a microplate reader-based detection method for the TAS cell migration assay, further enhancing its efficiency and applicability. Our research group previously developed the TAS assay to overcome the limitations of the widely used scratch assay, introducing advantages such as increased reproducibility, higher throughput, and reduced material consumption. By integrating microplate-based detection and Hoechst staining, we have now optimized the method for automated, high-throughput analysis, eliminating the need for a labor-intensive microscopy system. We also demonstrated its utility in assessing the effects of kinase inhibitors and MSC-derived EVs on cancer cell migration. These advancements position the TAS assay as a powerful and scalable tool for drug screening and cell migration studies, offering a robust alternative to conventional imaging techniques.

## 4. Materials and Methods

### 4.1. Cell Lines and Treatments

A549 (#CRM-CCL-185) human lung cancer cells were cultured in Dulbecco’s Modified Eagle Medium (Thermo Fisher Scientific, Waltham, MA, USA), LCLC-103H (#ACC-384) human lung cancer cells, HCT-116 (#CCL-247), green fluorescent protein (GFP)-expressing HCT-116, red fluorescent protein (RFP)-expressing HCT-116 (obtained from József Tóvári, National Institute of Oncology, Budapest, Hungary), and human colon cancer cells were cultured in Roswell Park Memorial Institute (RPMI) 1640 medium (Thermo Fisher Scientific) supplemented with a 10% heat-inactivated fetal bovine serum (FBS) (Invitrogen, Waltham, MA, USA) and a 1% penicillin and streptomycin (Merck, Kenilworth, NJ, USA) mixture under standard cell culture conditions (37 °C, humidified, 5% CO_2_). Mesenchymal stem cells (MSCs) were isolated from peritoneal dialysate of two individual pediatric patients treated with peritoneal dialysis at the Pediatric Center, Semmelweis University (31224-5/2017/EKU), as described previously [32,33], and cultured in Dulbecco’s modified Eagle’s medium/Nutrient Mixture F-12 (DMEM-F12, Thermo Fisher Scientific).

During the in vitro experiments, a dilution series (0–10 μM) of kinase inhibitors, including gefitinib, nintedanib, or sorafenib (Vichem Chemie Research Ltd., Budapest, Hungary), was used. Control cells were treated with an equivalent volume of DMSO (Merck). In addition, an alternative experimental setup involved treating the cells with extracellular vesicles (EVs; 0–7 × 10^9^ particles/mL) dissolved in phosphate-buffered saline (PBS). In both experiments, the cell culture medium contained 10% FBS.

### 4.2. Transient Agarose Spot (TAS) Assay

TAS assay was performed according to our previously described protocol [4]. Briefly, a 0.1% agarose (Merck) solution was prepared by boiling and stirring thoroughly, then stored at 70 °C in a thermoblock until use. Subsequently, 2 μL droplets were placed in the middle of the wells of a 96-well tissue culture plate (Sarstedt, Newton, MA, USA) and left at room temperature for 15 min to polymerise. Cells were seeded (n = 6–10 well/treatment group) at a density sufficient to reach near-full confluence (1.5 × 10^4^ cells/well A549, 7.5 × 10^3^ cells/well LCLC-103H, 2 × 10^4^ cells/well HCT-116), and incubated overnight to ensure proper attachment. Thereafter, the culture medium was removed, the agarose spots were gently aspirated from the bottom of the wells, and the cells were washed with sterile PBS. Finally, the treatment in question was applied for 72 h.

### 4.3. Cell Staining

Before data acquisition, various colorimetric and fluorescence-based cell staining methods were tested, with detailed staining protocols provided in Table 1. Kahle’s solution was prepared using 26% ethanol, 3.7% formaldehyde, and 2% glacial acetic acid.

### 4.4. Data Acquisition by Microscopy

Brightfield or fluorescence images of each well were captured at 0 and 72 h following the treatments using an Olympus IX81 microscope system (Olympus Corporation, Tokyo, Japan). Cell-free gap areas were manually annotated using a digitizer board and measured with ImageJ 1.48v software (National Institutes of Health, Bethesda, Rockville, MD, USA). The gap areas were finally calculated and presented as the percentage ratio (%) of the initial gap area.

### 4.5. Data Acquisition by Microplate Reader

Absorbance or fluorescence signal of each well was measured at 0 and 72 h, following the treatments using a CLARIOstar Plus microplate reader equipped with MARS v4.01 software (BMG Labtech, Ortenberg, Germany). During the measurements, matrix scanning mode was employed with a 30 × 30 matrix dimension focusing on a 4 mm diameter circular area in the center of each well, with a focal height set to 3.2 mm, choosing bottom optic for fluorescence detection. The optical settings for the various staining methods are provided in Table 2. The gain parameters were manually adjusted to ensure that the resulting fluorescence data fell within the upper third of the measurement range, allowing clear distinction between cell-covered and cell-free areas. Cell-free gap areas were annotated by excluding scan points above a manually defined threshold, followed by counting the number of scan points. The gap areas were finally calculated and presented as the percentage ratio (%) of the initial gap area.

### 4.6. Cell Viability Assay

Cell viability was assessed using MTT assay. At the end of the in vitro experiment, 10 μL of 5 mg/mL thiazolyl blue tetrazolium bromide (Merck), diluted in H_2_O, was added to each well (containing 100 μL culture medium) and incubated at 37 °C for 4 h. Subsequently, the supernatants were aspirated from the cells, and the intracellular crystals were dissolved with 100 μL of a DMSO-ethanol (1:1) mixture. The absorbance was recorded at 570 nm, with 690 nm used as the background in a CLARIOStar Plus microplate reader using MARS v4.01 software (BMG Labtech). The results were finally normalized and presented as the percentage ratio (%) of the control group values.

### 4.7. Mesenchymal Stem Cell-Derived Extracellular Vesicle Isolation and Characterization

EV1 and EV2 extracellular vesicles were isolated from the medium of the two primary MSC cultures derived from different patients on peritoneal dialysis. Cells were grown in T-175 cell culture flasks (Sarstedt) until reaching full confluence. The medium was then replaced with serum-free DMEM-F12 and incubated for 24 h. Afterward, the supernatant was separated, and cells and large EVs were removed by centrifugation at 400× *g* for 30 min and subsequent filtration using Filtropur S syringe filter (polyethersulfone, 0.2 μm pore size, Sarstedt). EVs were then purified using tangential flow filtration (polysulfone hollow fibers, 20 nm pore size, HansaBioMed Life Sciences Ltd., Tallinn, Estonia), followed by size-exclusion chromatography (70 nm qEV columns, Izon Science, Lyon, France).

EV characterization was performed in accordance with the current guidelines from the International Society for Extracellular Vesicles (ISEV) [34]. The nanoparticle size distribution was determined by nanoparticle tracking analysis (NTA) using ZetaView PMX-120 (Particle Metrix GmbH, Meersbush, Germany) with ZetaVIEW software 8.05.12 SP2 [35]. Surface-enhanced Fourier Transform Infrared Spectroscopy (SEIRS) measurements with in-house developed gold nanoparticles were carried out to analyze the protein and lipid content, employing a Varian 2000 spectrometer (Scimitar Series, Coral Springs, FL, USA) fitted with a diamond-attenuated total reflection cell (“Golden Gate” single-reflection attenuated total reflectance (ATR) unit, Specac, Orpington, UK) [36]. The morphology and structure of nanoparticles were analyzed using transmission electron microscopy (TEM), where samples were prepared on formvar-coated copper grids, stained with uranyl acetate, and examined using a Morgagni 268D electron microscope (FEI, Eindhoven, The Netherlands) [37,38]. The presence of specific exosome markers was investigated via immune-TEM, using anti-ALIX (#SAB4200477, Merck) and anti-CD81 (#SAB3500454, Merck) primary antibodies and anti-rabbit IgG gold-conjugated secondary antibodies (5 nm gold, #G7652, and 10 nm gold, #G7277, Merck). The images were captured with a JEOL 1011 microscope (Tokyo, Japan) [39].

### 4.8. Statistics

Statistical evaluation of the data was performed using GraphPad Prism 9.1.2 software (GraphPad Software Inc., San Diego, CA, USA). Pearson’s correlation was used for correlation analyses, and Brown–Forsythe and Welch ANOVA with Dunnett’s T3 test were applied for multiple comparisons. *p* ≤ 0.05 was considered statistically significant. Unless otherwise indicated, results are illustrated as mean ± SD of the corresponding groups. The applied tests, significances, and number of elements (n) are indicated in each figure legend.

## Figures and Tables

**Table 1:** Staining protocols.

	Fixation	Dye	Staining
SRB	10% TCA at 4 °C for 1 h	Sulforodamine B sodium salt (#S1402, Merck)	0.4% SRB in 1% AA for 30 min
FastGreen	Kahle’s solution at RT for 15 min	FastGreen FCF (#F7252, Merck)	0.1% FastGreen in 1% AA for 5 min
methylene blue	Kahle’s solution at RT for 15 min	Methylene blue solution acc. To Loeffler (#42335, Molar Chemicals, Hálasztelek, Hungary)	1% methylene blue in H_2_O for 5 min
Cresyl Violet	Kahle’s solution at RT for 15 min	Cresyl Fast Violet—Certistain (#K2247947, Merck)	0.14 mg/mL in 6.8 mM sodium acetate + 83 mM AA mix for 20 min
DiI	-	1,1′-Dioctadecyl-3,3,3′,3′-Tetramethylindocarbocyanine Perchlorate (#D282, Thermo Fisher Scientific)	0.1 mg/mL in cell culture medium for 24 h
WGA	-	Wheat Germ Agglutinin Alexa Fluor 488 conjugate (#W11261, Thermo Fisher Scientific)	0.01 mg/mL in cell culture medium for 4 h
Calcein AM	-	Calcein AM (#C3099, Thermo Fisher Scientific)	10 μM in cell culture medium for 1 h
Cell Tracker	-	Cell Tracker Green CMFDA (#C7025, Thermo Fisher Scientific)	5 μM in cell culture medium for 1 h
Hoechst	-	Hoechst 33,342 trihydrochloride (#B2261, Merck)	0.5 μg/mL (1:10 000) in cell culture medium for 4 h
PI	Kahle’s solution at RT for 15 min	Propidium Iodide Staining Solution (#51-66211E, BD Pharmingen, San Diego, CA, USA)	0.5 μM in cell culture medium for 5 min

Abbreviations: TCA: trichloroacetic acid; AA: acetid acid; RT: room temperature.

**Table 2:** Optical settings for data acquisition by microplate reader using various staining methods.

Staining	Measurement Method	Excitation [nm]	Emission [nm]
-	Absorbance	285	-
SRB	Absorbance	565	-
	Fluorescence	550	605
FastGreen	Absorbance	624	-
methylene blue	Absorbance	668	-
Cresyl Violet	Absorbance	590	-
	Fluorescence	583	627
DiI	Fluorescence	538	582
WGA	Fluorescence	470	515
Calcein AM	Fluorescence	490	533
Cell Tracker	Fluorescence	470	515
Hoechst	Fluorescence	355	455
PI	Fluorescence	550	605

## Data Availability

Data is contained within the article.

## Abbreviations

The following abbreviations are used in this manuscript:

EV	extracellular vesicle
FBS	fetal bovine serum
GFP	green fluorescent protein
MSC	mesenchymal stem cell
NTA	nanoparticle tracking analysis
RFP	red fluorescent protein
TAS	transient agarose spot
TEM	transmission electron microscopy
